# Microarray-Based Approach Identifies Differentially Expressed MicroRNAs in Porcine Sexually Immature and Mature Testes

**DOI:** 10.1371/journal.pone.0011744

**Published:** 2010-08-18

**Authors:** Lifan Luo, Lianzhi Ye, Gang Liu, Guochao Shao, Rong Zheng, Zhuqing Ren, Bo Zuo, Dequan Xu, Minggang Lei, Siwen Jiang, Changyan Deng, Yuanzhu Xiong, Fenge Li

**Affiliations:** Key Laboratory of Pig Genetics and Breeding of Ministry of Agriculture, and Key Laboratory of Agricultural Animal Genetics, Breeding and Reproduction of Ministry of Education, Huazhong Agricultural University, Wuhan, People's Republic of China; University of Western Cape, South Africa

## Abstract

**Background:**

MicroRNAs (miRNAs) are short non-coding RNA molecules which are proved to be involved in mammalian spermatogenesis. Their expression and function in the porcine germ cells are not fully understood.

**Methodology:**

We employed a miRNA microarray containing 1260 unique miRNA probes to evaluate the miRNA expression patterns between sexually immature (60-day) and mature (180-day) pig testes. One hundred and twenty nine miRNAs representing 164 reporter miRNAs were expressed differently (p<0.1). Fifty one miRNAs were significantly up-regulated and 78 miRNAs were down-regulated in mature testes. Nine of these differentially expressed miRNAs were validated using quantitative RT-PCR assay. Totally 15919 putative miRNA-target sites were detected by using RNA22 method to align 445 NCBI pig cDNA sequences with these 129 differentially expressed miRNAs, and seven putative target genes involved in spermatogenesis including *DAZL*, *RNF4* gene were simply confirmed by quantitative RT-PCR.

**Conclusions:**

Overall, the results of this study indicated specific miRNAs expression in porcine testes and suggested that miRNAs had a role in regulating spermatogenesis.

## Introduction

microRNAs (miRNAs) are small non-coding RNAs (typically 19–23 nucleotides) that play important roles in regulating posttranscriptional translation. The first discovered miRNA, lin-4, is involved in developmental timing in the *nematode C. elegans* [1]. To date, 10883 miRNA sequences have been published on the Sanger miRNA Registry (http://www.sanger.ac.uk/software/Rfam/mirna, miRbase Release 14.0). They are increasingly being shown to play vital roles in spermatogenesis, muscle development, feed intake and other important physiological process [2]–[4]. Such as the myostatin allele in muscle mass QTL interval is characterized by a G to A transition in the 3′ un-translated region (UTR) that creates a target site for miR-1 and miR-206 which are highly expressed in skeletal muscle. This causes translational inhibition of the myostatin gene and hence contributes to the muscular hypertrophy of Texel sheep [5].

Spermatogenesis is a complex process through which diploid germ cells proliferate and differentiate into haploid spermatozoa [6]. It is estimated that about 1000 genes involved in spermatogenesis, and 351 of these genes appear to be expressed only in the male germs [7]. A large number of genes are expressed at grossly higher levels in meiotic and/or early haploid spermatogenic cells than in somatic cells, yet they too are translated inefficiently [8]. Such repression could be reached by ribosomal protein binding with target genes or by some translational control elements which could be bound at 3′ UTRs of target genes [9]. miRNAs are a large family of small regulatory elements that direct messenger RNA degradation or disrupt mRNA translation by binding the UTRs and coding sequences (CDS) of target mRNAs [10], [11]. For example, miR-122a was suggested targeting a reporter mRNA containing sequences from the 3′-UTR of the transition protein 2 (*TNP2*), a post-transcriptionally regulated testis-specific gene involved in chromatin remodeling during mouse spermatogenesis [2]. The over-expression of miR-34c in HeLa cells led to a shift of the expression profile toward the germinal lineage, and miR-34c could play a role in the late steps of spermatogenesis [12].

Presently many efforts have been made to the discovery of gonad-expressed miRNAs. By using a new small RNA cloning method, 141 mouse testis miRNAs were isolated, of which 28 miRNAs showed the highest expression levels in meiotic (pachytene spermatocytes), or haploid (round and elongated spermatocytes) germ cells, suggesting that late meiotic and haploid germ cells are the main source of miRNA production during spermatogenesis [13]. About 54 porcine miRNAs have been identified, however their expression and function in the porcine germ cells are still poorly understood [14]–[16]. In addition, it is necessary to study porcine miRNAs due to the increasing interest in pig genetics and the benefits of using the domestic pigs as a model for the study of human male infertility. Therefore, we investigated differentially expressed miRNAs between immature and mature testis tissues of Large White boars by miRNAs array analysis, and predicted target genes of these miRNA and analyzed the relationship between those putative genes and spermatogenesis.

## Results and Discussion

### miRNA microarray analysis

A custom-made mammalian miRNA microarray was used to evaluate the expression of porcine miRNAs. At the design time of the microarray, there were 2522 mammal mature miRNAs including 711 human, 658 mice, 348 rat and 54 porcine mature miRNAs. After removing the redundant sequences, there remained 1260 unique mature miRNA sequences (See probe list of the microarray in Table S1). The microarray contained 1260 probes complementary to these sequences, and all probes were repeated triplicate in one microarray. Microarray hybridization with RNA samples prepared from three 60-day (sexually immature) porcine testes and three 180-day (mature) porcine testes detected expression of 704 miRNAs, with 449.67±102.66 miRNAs per sample (Table S2). Of the 704 detectable miRNAs, miRNAs count present in all six samples was 261 (20.71%), which was a little lower than previously reported (28.3%) [17]. Thirty of 54 porcine miRNAs in miRbase release 10.0, were detected (25 in 60-day testes and 27 in 180-day testes).

The microarray data showed that several microRNAs including let-7, miR-923, miR-202, miR-21 and miR-145 were highly expressed in the porcine testes (Table S2). Pairwise significance analysis indicated 129 expressed miRNAs representing 164 miRNAs probes had changed expression profiles between 60-day and 180-day testis samples (p≤0.1), and in 180-day (mature) porcine testes down-regulated expression appeared in 78 miRNAs while up-regulated expression appeared in other 51 miRNAs (Table S3). Among 129 differentially expressed miRNAs in pig testes, 8 miRNAs including mmu-let-7e and mmu-miR-181b shared the same expression profile as the previous study in sexually immature/mature mouse testes [18], and 10 miRNAs including hsa-miR-1 and mmu-miR-709 had the same expression profile in the immature/mature rhesus monkey testes [19]. Five miRNAs (mmu-miR-449, rno-miR-34b, mmu-miR-34c hsa-miR-181d, mmu-miR-214) appeared to be differentially expressed in two independent reports [18], [19]. Another four miRNAs (mmu-miR-34b, mmu-miR-122a, mmu-miR-16 and mmu-miR-101) were confirmed to be differentially expressed in mouse testes by using conventional Northern blot analysis [2].

Mature miRNA sequences are highly conserved in different animal species and only 1–3 nucleotide differences. For example, there are only 1–2 nucleotide differences in the miR-181b respectively from *Monodelphis domestica*, *Gorilla*, *Bos taurus* and three members from *Homo sapiens*. Thus, cross-hybridization among members from the same family and among orthologs across species could explain these microarray results. All above four orthologs of miR-181b (m, g, b, and h) were up-regulated in the 60-day testis (Table S2).

### microRNA expression validation

**Figure 1:**
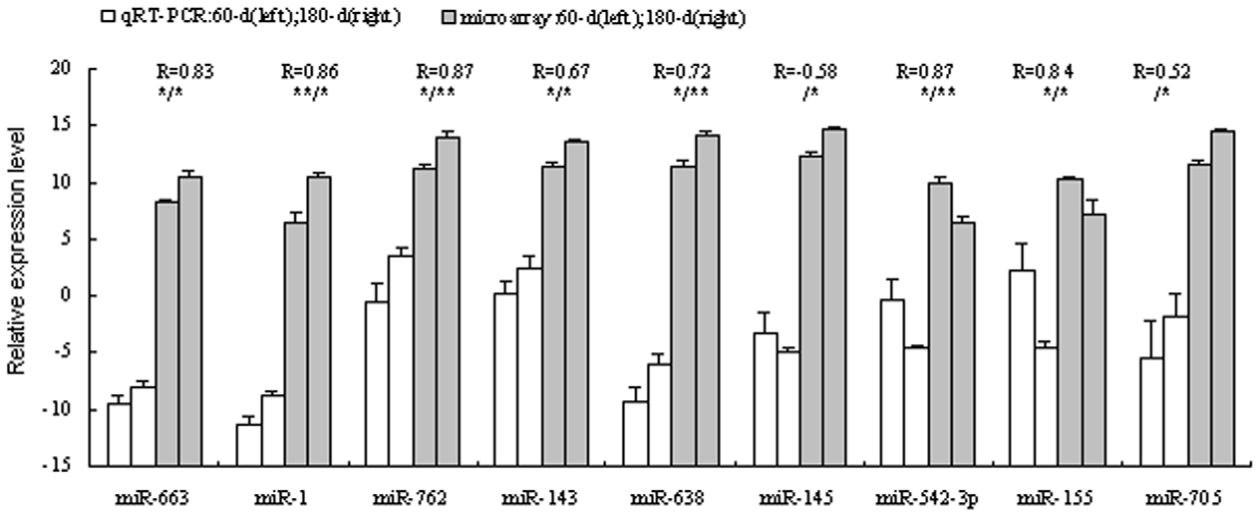
Validation of the microarray results using qRT-PCR method. The X-axis represents the miRNAs and the Y-axis shows the relative expression levels of miRNAs (-ΔC_t_ values for qRT-PCR; Log(sample signal, 2) for microarray). The number of biological replicates is three for both assays. R represents the Pearson correlation coefficient. The significance of differences for the expression between 60-d (immature, 60-day) and 180-d (mature, 180-day) testes was calculated using two-tailed T-test. *, p≤0.05; **, p≤0.01 (left for qRT-PCR, and right for microarray).

In order to validate the DNA microarray results, real-time RT-PCR with commercially available primers (Table S4) was carried out separately to investigate the differential expression pattern of 9 miRNAs - miR-663, miR-1, miR-762, miR-143, miR-638, miR-145, miR-542-3p, miR-155 and miR-705. Our results showed a high level of concordance between these two methods in 8 of the 9 comparisons (Pearson correlation coefficient ≥0.52, Figure 1). miR-705 has a high Pearson correlation coefficient of 0.52, but a significant difference between immature and mature testes has not been observed using qRT-PCR. And a contradiction expression profile was detected in miR-145. The expression levels varied dramatically, and the variance probably came from biological differences between the samples.

### Differentially expressed miRNA character analysis

Using a homology search based on genomic survey sequence analysis and microRNA (miRNA) secondary structure, a total of 72 porcine differentially expressed miRNAs were identified and then were located in pig genome covering all chromosomes except SSC14 and SSCY, and 14 (19.44%) miRNAs were mapped to SSCX (Table S5). One reason is that SSCY sequences have not been released yet, and the other is that miRNAs are scarcely located to SSCY. No miRNA was found on the Y chromosome in any species and the densities were greater than twofold those on autosomes in seven of eight mammalian species (p<0.01) [20]. These X-linked miRNAs were expressed in a testis-preferential or testis-specific pattern, suggesting that they have functional roles during spermatogenesis, including the possibility that they contribute to the process of meiotic sex chromosome inactivation, or that they may be essential for post-transcriptional regulation of autosomal mRNAs during the late meiotic and early postmeiotic stages of spermatogenesis [21].

### Putative miRNA target gene prediction and expression array of target genes

To gain insight into the function of these microRNAs, 445 cDNA sequences were obtained from NCBI GenBank database (Accession numbers were listed in Table S6). Totally 15919 miRNA-mRNA interaction sites for 129 differentially expressed miRNAs had found within the full-length cDNA sequences, and 787, 12250, 2882 were located in 5′UTR, CDS and 3′UTR, respectively (Table S6). Most (76.95%) of these binding sites were located to CDS because the UTR sequences had not been fully identified. It is estimated that there is about 19977 genes (http://www.ensembl.org/Sus_scrofa/) and at least 1200 porcine miRNAs which was deduced from the number of human (1240 mature human miRNAs in miRbase Release 15.0), and there will be 6.65 million, 0.33 million, 5.11 million, 1.20 million miRNA-target sites in full-length, 5′UTR, CDS and 3′UTR, respectively. Averagely, each gene has 35.66 miRNA-target sites and 25.28 target miRNAs. Three genes, NOTCH4, C4, NCOA2, assigned the highest number of miRNA interaction sites. NOTCH4 is a unique member of the NOTCH family, which can affect the implementation of differentiation, proliferation, and apoptotic programs, influencing organ formation and morphogenesis [22]. Complement component 4 (C4) is the only component coded for by 2 nearly isotypic genes, C4A and C4B. *C4* genes are located near MHC region and expected to play a key role in immunity [23]. *NCOA2* gene was previously identified as a transcriptional activator for steriod receptors and nuclear receptors [24]. GO and KEGG analysis showed that these genes are involved in the life process including multi-organism process, reproductive process and reproduction, and play important roles in 17 pathways (Table S7). Amony 129 differentially expressed miRNAs, miR-762, miR-149* and miR-663 were the top 3 highest number of miRNA interaction sites.

**Figure 2:**
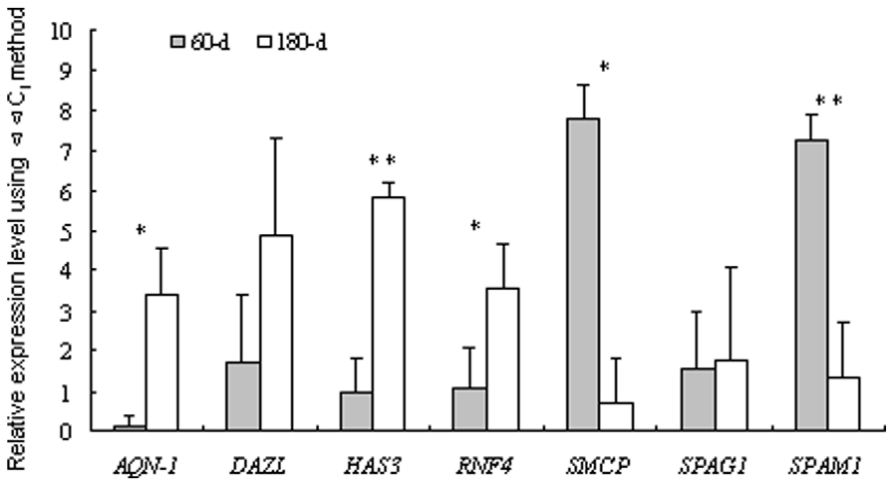
miRNA putative target genes expression in porcine testis was identified by qRT-PCR. The X-axis represents the specific gene and the Y-axis shows the ΔΔC_t_ values of genes. ΔΔC_t_ value has a negative relationship with the gene expression level, so the smaller ΔΔC_t_ value has a higher expression level. The number of biological replicates is three. The significance of differences for the expression between 60-d (immature, 60-day) and 180-d (mature, 180-day) testes was calculated using two-tailed T-test. *, p≤0.05; **, p≤0.01.

Real-time RT-PCR was used to validate the target genes which are associated with mammalian testis development and spermatogenesis. The results showed that in mature testes: *AQN-1*, *HAS3*, *RNF4* gene were down regulated while *SMCP* and *SPAM1* gene were up regulated (p≤0.05); DAZL and *SPAG1* had a tendency to be down-regulated, and up-regulated, respectively (Figure 2, ΔΔC_t_ value has a negative relationship with the gene expression level). By comparing the expression profiling of miRNAs and target genes, only about half of these putative target genes remained due to the negative relationship of the expression patterns between miRNA and its target mRNA (Table 1). One of these putative target genes is deleted in azoospermia like gene (*DAZL*), targeted by miR-34b, miR-34c *etc*. In mice, disruption of the *DAZL* gene leads to loss of germ cells and complete absence of gamete production [25]. An A386G (T54A) mutation occurring in the RNA-binding domain of the DAZL protein has been associated with susceptibility to spermatogenic failure in the Taiwanese [26], and it was associated with the female reproductive traits in pigs [27]. Predictions associated small nuclear RING finger protein RNF4 (*SNURF*) gene with miR-638, miR-705 and miR-762 *etc*. RNF4 possesses a C-terminal RING finger and acts as a transcription regulator. RNF4 expressed more abundantly in murine adult testis [28]. In adult rat testis, RNF4 mRNA and protein accumulate in postmeiotic round and elongating spermatids, suggesting that this protein is involved in the regulation of processes required for late steps of spermatid maturation, during which vast amount of protein degradation and chromatin compaction take place [29], and it was associated with the female reproductive traits in pigs [30].

**Table 1:** Number of binding miRNAs of seven potential target genes involved in spermatogenesis.

NO. Binding miRNAs	*AQN-1*	*DAZL*	*HAS3*	*RNF4*	*SMCP*	*SPAG1*	*SPAM-1*	Total
Bioinformatics	25	14	55	20	17	19	29	179
Bioinformatics + Microarray Array + qPCR	14	10	25	12	7	9	13	90
Positive percentage	0.56	0.714	0.455	0.6	0.412	0.474	0.448	0.503

Recently, a novel class of 26–30 nt RNAs (piRNAs) has been described in the testis, where they bind a spermatogenesis-specific protein belonging to the Argonaute protein family called PIWI. Like termed germline small RNAs (gsRNAs), miRNA and short interference RNA and other small RNAs expressed in testes, piRNAs are thought to be involved in gene silencing [31], [32]. However, piRNAs are apparently present at low levels in mature spermatozoa, and piRNA sequences are not conserved between species [31], [32] The majority of piRNAs are antisense to transposon sequences, suggesting that transposons are the piRNA target [33], but its expression profiles, and function of piRNAs are still poorly understood.

## Materials and Methods

### Ethics statement

All research involving animals were conducted according to the regulation (No. 5 proclaim of the Standing Committee of Hubei People's Congress) approved by the Standing Committee of Hubei People's Congress, P. R. China. Sample collection was approved by the ethics committee of Huazhong Agricultural University (No. 30700571 for this study).

### Animals and RNA extraction

Three young Large White boars of 60 days (sexually immature) and 3 of 180 days (sexually mature defined according to the reference [34]) Large White boars were obtained from the pig farm of Huazhong Agricultural University (Wuhan, China). Whole testes were removed from animals and were immediately snap-frozen in liquid nitrogen and stored at −80°C. Total RNAs were extracted by Trizol reagent (Invitrogen). All the procedures were carried out according to manufacturer's protocols.

### µParaflo™ microRNA microarray assay

The custom-made microarray contained 1260 unique miRNA probes generated from 2522 mammalian miRNAs including 711 human, 658 mice, 348 rat and 54 porcine miRNAs (Table S1), and all of the oligonucleotide probes were repeated triplicate in one microarray. MicroRNA microarray analysis was performed by LC Sciences (Houston, TX). Briefly, the assay started from 2 to 5 µg total RNA sample, which was size fractionated using a YM-100 Microcon centrifugal filter (from Millipore) and the small RNAs (<300 nt) isolated were 3′-extended with a poly(A) tail using poly(A) polymerase. An oligonucleotide tag was then ligated to the poly (A) tail for later fluorescent dye staining; two different tags were used for the two RNA samples in dual-sample experiments. Hybridization was performed overnight on a μParaflo™ microfluidic chip using a micro-circulation pump (Atactic Technologies). On the microfluidic chip, each detection probe consisted of a chemically modified nucleotide coding segment complementary to target microRNA (from miRBase release 10.0, 1260 miRNA probes in total) or control RNA and a spacer segment of polyethylene glycol to extend the coding segment away from the substrate. Multiple control probes including BKG, PUC2PM-20B, PUC2MM-20B and 5S-rRNA, were used for quality controls of chip production in each chip.

The detection probes were made by *in situ* synthesis using PGR (photogenerated reagent) chemistry. The hybridization melting temperatures were balanced by chemical modifications of the detection probes. Hybridization used 100 μL 6× SSPE buffer (0.90 M NaCl, 60 mM Na_2_HPO_4_, 6 mM EDTA, pH 6.8) containing 25% formamide at 34°C. After hybridization detection used fluorescence labeling using tag-specific Cy3 and Cy5 dyes. Microarray experiments were performed three times using biological samples. Hybridization images were collected using a laser scanner (GenePix 4000B; Molecular Devices, Sunnyvale, CA, USA) and digitized using the Array-Pro image analysis software (Media Cybernetics, Bethesda, MD, USA). Data were analyzed by first subtracting the background and then normalizing the signals using a LOWESS filter (Locally-weighted Regression). A transcript to be listed as detectable must meet at least two conditions: signal intensity higher than 3× (background standard deviation) and spot CV <0.5, and CV was calculated by (standard deviation)/(signal intensity) [17]. When repeating probes were present on an array, a transcript was listed as detectable only if the signals from at least 50% of the repeating probes are above detection level. Furthermore, “bad spots” that had signal values deviated more than 50% of average values of repeating spots and/or spot CV larger than 0.5 were discarded. For two color experiments, the ratio of the two sets of detected signals (log_2_ transformed, balanced) and p-values of the t-test were calculated. Differentially detected signals were those with a slightly relaxed p-value cutoff of 0.1 [35], [36], in case that the true positives were excluded.

### Quantitative real-time RT-PCR to validate miRNA expression

The microarray findings were validated using quantitative real-time RT-PCR. Ten nanogram RNAs were 3′-extended with a poly (A) tail using poly (A) polymerase, and then reverse transcribed to cDNA using the RT-PCR System (Promega). The expression levels of miRNAs were detected in 60-day porcine testes and 180-day porcine testes by SYBR Green I assay (TOYOBO). Each real-time PCR (in 25 µL) included 12.5 µL SYBR Green Real-time PCR Master Mix, 350–500 ng cDNA, 0.3 µM primers (Table S4). The cycling conditions consisted of 1 cycle at 95°C for 3 min, followed by 40 cycles at 94°C for 20 sec, 58°C for 20 sec, and 72°C for 10 sec, with fluorescence acquisition at 74°C in single mode. The specific PCR products were confirmed by the results of melting curve analysis and agarose gel electrophoresis. cDNAs from three testis samples at each stage were used as template to detect the expression changes of the miRNAs, and all PCRs were performed in triplicate. The miRNA was considered to be undetectable when its *C*_t_ value exceeded 35 in the sample tissue. miRNA expression levels were quantified relatively to the expression of 18S RNA using Gene Expression Macro software (Bio-Rad, Richmond, CA, USA) by employing Δ*C*_t_ value. Student's *t*-test was conducted to identify differentially expressed miRNAs. Due to the negative relationship between C_t_ and expression level, an improved method of the previous report [37] was used to compare the results of qRT-PCR and microarray by plotting the -Δ*C*_t_ values of qRT-PCR versus the log2 of the microarray signal for each miRNA.

### Bioinformatics analysis

A homology search based on genomic survey sequence analysis and human miRNA secondary structure was used to map the miRNA genes. Human miRNA and its corresponding pri-miRNA sequences were obtained from the Sanger miRNA Registry (http://www.sanger.ac.uk/software/Rfam/mirna). To retrieve homologous pig miRNA genes, the human pri-miRNA sequence was used as a query sequence to search homology using BLASTN (Basic Local Alignment Search Tool-nucleotide) on the NCBI pig sequence database with HTGS or Trace-WGS options. The porcine miRNAs were predicted to be located in the corresponding target contig or shot-gun sequences, if the similarity was larger than 80%.

To fully inverstgate the function of the differentially expressed miRNAs, we collected pig cDNA sequences were randomly selected from GenBank and performed a GO term and KEGG pathway annotation using the DAVID gene annotation tool (http://david.abcc.ncifcrf.gov/). Here the “Full-Length” cDNA sequences were used to match with the differentially expressed miRNAs sequence by the RNA22 [38] to predict the putative target genes and corresponding target sites, since target mRNAs could be repressed as efficiently by miRNA-binding sites in the 5′ UTR and CDS as in the 3′ UTR [10], [11].

### Putative target gene validation

Seven candidate target genes involved in spermatogenesis, including *AQN-1*, *DAZL*, *HAS3*, *RNF4*, *SMCP*, *SPAG1* and *SPAM1* genes, were confirmed by real time RT-PCR. From each sample, 10 mg of total RNA was incubated with two units of RNase-free DNase I (New England BioLabs, Inc.) to remove DNA contamination from RNA. RNAs were 3′-extended with a poly (A) tail, then the first cDNA strand was synthesized and used as template for quantitative real-time RT-PCR with gene specific primers (Table S4) and *β-actin* was selected as the endogenous reference. All PCRs were performed in triplicate and gene expression levels were quantified relatively to the expression of *β-actin* using Gene Expression Macro software (Bio-Rad, Richmond, CA, USA) by employing an optimized comparative *C*_t_ (ΔΔ*C*_t_) value method. Student's *t*-test was conducted to identify differentially expressed miRNAs by comparing ΔΔ*C*_t_ value of two groups [39].

## Supporting Information

Table S1 All the miRNAs probes used in this research. 2522 mammal mature miRNAs were included. After removing the redundant sequences, 1260 unique mature miRNA sequences remained. The signs (*, 3p, 5p and additional letters) of miRNA names are explained at http://www.mirbase.org/help/nomenclature.shtml.
(0.21 MB XLS)

Table S2 Average signal of detectable transcripts. Detectable transcripts must meets at least two conditions: signal intensity higher than 3× (background standard deviation) and spot CV <0.5. CV is calculated by (standard deviation)/(signal intensity).
(0.16 MB XLS)

Table S3 Differentially expressed miRNAs detected in porcine sexually immature and muture testes tissues (p<0.1). p-value with red, yellow and blue means p<0.01, p<0.05 and p<0.1, respectively.
(0.04 MB XLS)

Table S4 Primer pairs used to confirm the differentially expressed miRNAs and target genes.
(0.02 MB XLS)

Table S5 Characteristics analysis of differentially expressed miRNAs.
(0.12 MB XLS)

Table S6 The results of 445 gene cDNA sequence aligned with 129 differentially expressed (DE) miRNAs by RNA22. Part 1: miRNA-mRNA interaction site counts and binding DE miRNAs counts of each potential target gene. Part 2: miRNA-mRNA interaction site counts and target gene counts of each DE miRNA.
(0.50 MB XLS)

Table S7 GO Functional Enrichment and KEGG pathway annotation of the miRNA potential targets.
(0.03 MB XLS)
